# Evaluation of sonographic and clinical measures in early versus late third trimester for birth weight prediction

**DOI:** 10.1002/ijgo.15911

**Published:** 2024-09-13

**Authors:** Shira Regev‐Sadeh, Wisam Assaf, Adi Zehavi, Nadav Cohen, Ofer Lavie, Ariel Zilberlicht

**Affiliations:** ^1^ Tel Aviv University Faculty of Medicine Tel Aviv Israel; ^2^ Department of Obstetrics and Gynecology Lady Davis Carmel Medical Center Haifa Israel; ^3^ Rappaport Faculty of Medicine Technion‐Israel Institution of Technology Haifa Israel

**Keywords:** diabetes, fetal growth charts, fetal weight assessment, large for gestational age, macrosomia, obesity, small for gestational age

## Abstract

**Objective:**

To evaluate the optimal timing for fetal weight estimation during the third trimester.

**Methods:**

This retrospective cohort study involved fetal weight estimations from both early (28^+0^–36^+6^ weeks) and late (37^+0^ weeks and beyond) third trimester. These estimations were converted to predicted birth weights using the gestation‐adjusted projection formula. Birth weight predictions were compared with actual birth weights, to identify the most effective timing for weight prediction.

**Results:**

The study included 3549 cases, revealing mean percentage errors (MPE) of −3.69% for early sonographic assessments, −2.5% for late sonographic assessments, and −1.9% for late clinical assessments. A significant difference was found between early and late sonographic estimations (*P* < 0.001), whereas late sonographic and clinical assessments did not differ significantly (*P* = 0.771). Weight predictions for fetuses below the 10th and above the 90th centiles were less accurate than for those within the 10th–90th centiles (*P <* 0.001). In women with obesity, late clinical estimations were less precise (MPE of −5.85) compared with non‐obese women (MPE of −1.66, *P* < 0.001). For women with diabetes, early sonographic estimations were more accurate (MPE of −1.31) compared with non‐diabetic patients (MPE of −3.94, *P* < 0.001) though this difference did not persist later in pregnancy.

**Conclusion:**

Sonographic and clinical weight predictions in the late third trimester were more accurate than earlier third‐trimester sonographic assessments, hence continuous follow up and assessments closer to term are important. In women with diabetes, no adjustments in weight prediction methods are necessary. Accurately predicting birth weights for abnormally small or large fetuses remains challenging, indicating the need for improved screening and diagnostic strategies.

## Synopsis

Late third‐trimester sonographic and clinical fetal weight predictions are more accurate than early estimations, with special considerations needed for specific maternal and fetal subpopulations.

## 1 INTRODUCTION

Fetal weight measurements play a key role in antenatal assessment, affecting both timing and method of delivery.^1^ Previous studies have shown mean percentage errors (MPE) in ultrasound weight prediction of 5%–10% when compared with actual birth weights.^2^, ^3^ In special populations of abnormally small and large fetuses, the accuracy varies with MPE of up to 15%.^4^, ^5^, ^6^, ^7^ Sonographic fetal assessment at full term carries several limitations, including fetal head fixation in the maternal pelvis after engagement, which may disturb head dimensions, and occasionally decreased amniotic fluid volume, which may limit the accuracy of measurements. In contrast, birth weight estimations conducted within a short interval from labor potentially eliminate the effect of fetal growth from the time of ultrasound measurement until birth.^8^, ^9^

Considering the technical limitations of fetal weight assessment at term, and assuming that normal fetuses will not cross growth curve centiles, Mongelli and Gardosi^10^ proposed a gestation‐adjusted projection formula to predict birth weights based on sonographic examinations carried out earlier during pregnancy. Using the projection formula, several studies have aimed to evaluate the optimal timing for prediction of birth weight by ultrasound during the third trimester, but results remain inconclusive.^11^, ^12^, ^13^, ^14^ Therefore, the aim of the present study is to assess the most favorable timing for fetal weight estimation during the third trimester, on a large cohort of women from the Israeli population, using locally developed growth charts, with the purpose of predicting birth weight.

## 2 MATERIALS AND METHODS

### 2.1 Study population

This was a single‐center retrospective cohort study, including initially all women who had undergone vaginal and cesarean deliveries at our center between January 2015 and September 2020. Inclusion criteria were women with singleton gestations, who had documented sonographic estimated fetal weights (EFW) in both early (28^+0^–36^+6^ weeks) and late (37^+0^ weeks and beyond) third trimester and who delivered at 37 weeks or beyond. Exclusion criteria included patients without documented fetal weight estimations at both time periods and cases of intrauterine fetal demise.

### 2.2 Data collection

Clinical data were collected from patients’ electronic medical records and included maternal age and gestational age at delivery, maternal body mass index at first visit and delivery, gestation formula, mode of delivery, maternal comorbidities (gestational diseases including gestational and pregestational diabetes, hypertensive disorders and others, and pre‐existing background diseases). Sonographic EFW at early and late third trimester (including gestational ages during sonographic assessment), clinical EFW at late third trimester if conducted, fetal gender, and actual birth weights were also recorded.

Early sonographic measurements were recorded in antenatal clinics, as part of routine obstetric follow up, complying with the accepted methods for EFW. Late sonographic and clinical assessments were conducted either in antenatal clinics or upon admission to hospital, up to 2 weeks before delivery. All ultrasound EFWs were calculated based on biometric data using Hadlock's formula.^15^

### 2.3 Statistical analysis

Predicted birth weights were calculated using the gestation‐adjusted projection method^10^ based on sonographic measurements at both early and late third trimester and clinical estimations during the late third trimester using locally developed growth charts.^16^ Predicted birth weights were compared with actual birth weights and MPE were calculated and compared using Wilcoxon signed rank test for paired variables. A two‐sided *P* value less than 0.05 was considered statistically significant. The data were processed and analyzed with the software SPSS version v29 (IBM, Armonk, NY, USA).

The study was approved by Carmel Medical Center's Institutional Review Board, approval number CMC‐0113‐23. Due to the retrospective nature of the study, patients’ consent was not required.

## 3 RESULTS

Among 19 917 deliveries that occurred in our center throughout the study period, 3549 (17.8%) cases met all the inclusion criteria and were therefore included in the study. Clinical weight estimations, which were assessed at the same time in pregnancy as the late sonographic estimations, were available in 928 (26.1%) cases. Median gestational ages at early and late third trimester in which weight estimations were conducted were 32^+2^ and 39^+1^ weeks, respectively. Median gestational age at birth was 39^+4^ weeks (Table 1).

**TABLE 1:** Patient characteristics.^a^

Maternal characteristics	*n*	%
Background disease	702	19.8
Maternal gestational disease		
Pregestational/gestational diabetes	340	9.8
Hypertensive disorders^b^	116	3.3
Other^c^	23	0.6
Smoker	137	3.9
Obesity^d^	313	8.8
Mode of birth		
Cesarean section	458	12.9
Vaginal	3091	87.1

Maternal characteristics	Median	Range	Percentile^e^	Projected birth weight^e^
Maternal age, years	31	17–47		
Maternal BMI before pregnancy	22.52	13.22–51.12		
Parity	1	0–11		
Gestational age at early EFW, week	32^+2^	28^+0^–36^+6^		
Gestational age at late EFW, week	39^+1^	37^+0^–43^+0^		
Gestational age at birth, week	39^+4^	37^+0^–43^+2^		
Sonographic early EFW, g	1978		61.28	3409.20
Sonographic late EFW, g	3300		59.43	3379.96
Clinical late EFW, g	3400		64.62	3449.15
Birth weight, g	3300		52.37	

Abbreviations: BMI, body mass index (calculated as weight in kilograms divided by the square of height in meters); EFW, estimated fetal weight.

^a^ Data are presented as number (percentage) or as median (range) unless otherwise stated.

^b^ Chronic hypertension, gestation hypertension, pre‐eclampsia, superimposed pre‐eclampsia, eclampsia.

^c^ Gestation thrombocytopenia, hypothyroidism, intrahepatic cholestasis of pregnancy, toxoplasmosis, and cytomegalovirus infections.

^d^ BMI greater than 30.

^e^ Median centiles and projected birth weights are presented for each gestational period.

A notable difference was observed between projected birth weights obtained from early and late third trimester and actual birth weights (*P* < 0.001). Specifically, projected weights from early sonographic evaluations had overestimated the actual birth weight in 2210 (62%) cases, whereas estimation from late sonograms did so in 2122 (60%) cases. Projected weights from clinical weight estimations were larger than actual birth weights in only 518 (56%) cases.

**FIGURE 1:**
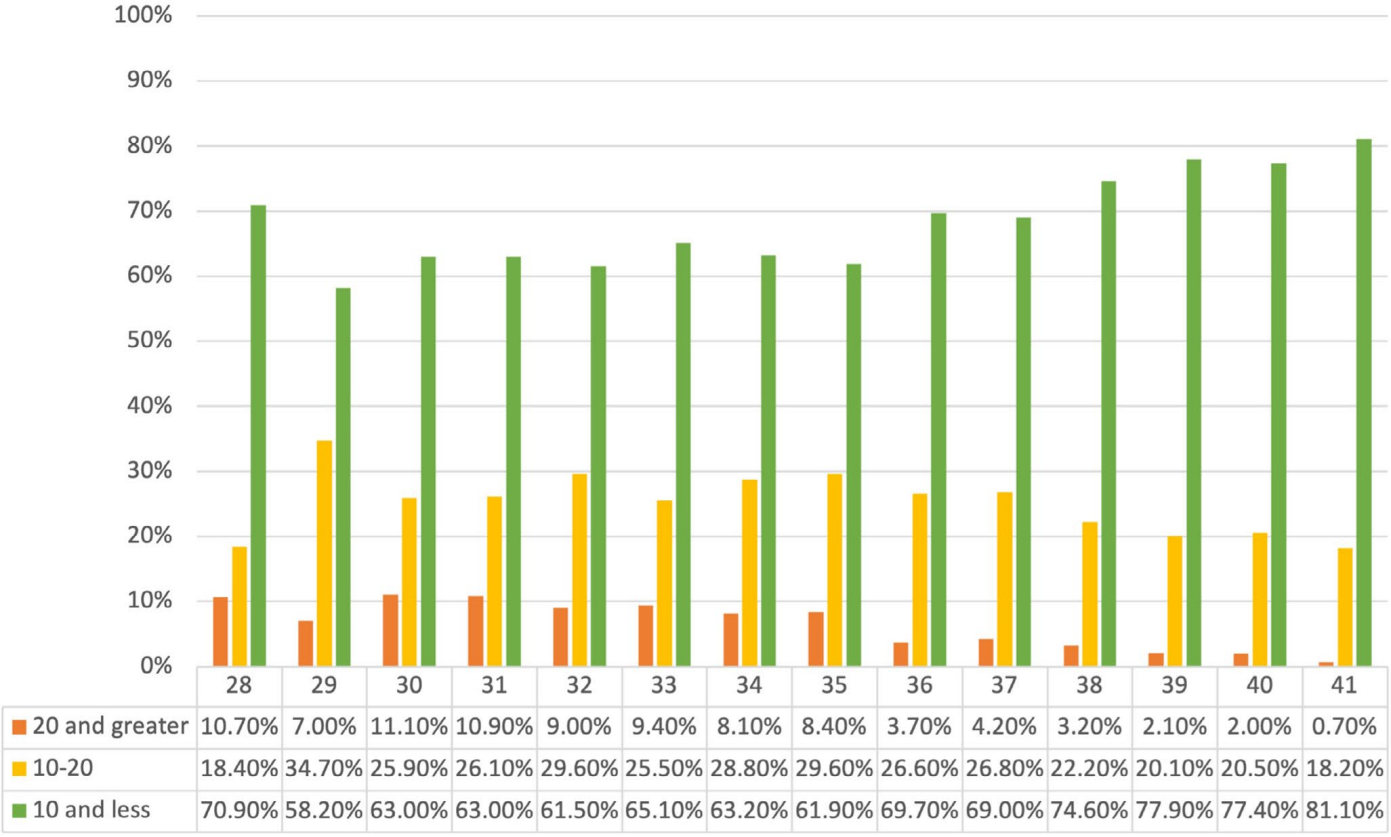
Accuracy of sonographic weight prediction by gestational week in relation to birth weight. Projected weights in each gestational week were calculated based only on sonographic weight estimations. Accurate weight predictions were defined as predictions within ±10% of actual birth weights.

Mean errors, percentage errors, and the proportion of projected weights within 10% and 20% in predicted birth weights from actual birth weights in the general study population are presented in Table 2. Weight estimations using sonography and clinical assessment during the late third trimester were found to be more accurate than sonographic predictions made in the early third trimester (*P* < 0.001). No significant difference was observed when comparing the MPE between late sonographic and clinical assessments (*P* = 0.771). Sonographic weight predictions for each individual gestational week were compared with actual birth weights (Figure 1). The largest proportion of accurate weight prediction within ±10% from the actual birth weight, was seen in weight estimations conducted during week 41 of pregnancy. Moreover, an upward trend in the proportion of accurate estimations is seen in weight predictions conducted in gestational weeks during the later third trimester, simultaneous to a downward trend in predictions larger than 10% and 20%.

**TABLE 2:** Measures of accuracy in birth weight prediction in the general study population.^a^

Prediction	Mean error (g) ± 95% CI^b^	MPE	Random error	MPE ± 95% CI^b^, ^c^	Mean absolute percentage error	Predictions (%) within ±10%	Predictions (%) within ±20%
Early sonographic projected weight	–92 ± 733.54	−3.69	11.73	−3.69 ± 22.99	9.32	64.47	93.52
Late sonographic projected weight	−64 ± 543.65	−2.5	8.54	−2.5 ± 16.74	7	75.57	97.38
Late clinical projected weight	−42 ± 566.42	−1.92	8.85	−1.92 ± 17.35	6.97	76.50	96.66

Abbreviations: CI, confidence interval; MPE, mean percentage error.

^a^ The mean deviation from actual birth weight is presented as the mean error in grams ±95% confidence interval (CI). The systematic error is the MPE of the projected weights, calculated as the mean of (birth weight – sonographic or clinical projected weight)/birth weight × 100. Negative values represent overestimation of actual birth weight and positive values represent underestimations. Random error is the standard deviation of the mean percentage error. Predictions within ±10% and ±20% are the proportion of calculated projected weights within 10% and 20% of the actual birth weights. All projected weights from weight estimations were compared with actual birth weights using Wilcoxon signed ranks test, all values were significantly different (*P* < 0.001).

^b^ According to the limits of agreement method.

^c^ MPE were compared using Wilcoxon signed ranks test. MPE of early and late sonographic weight estimations were significantly different (*P* < 0.001); no significant difference was shown between MPE of late sonographic and clinical estimations (*P* = 0.771).

**FIGURE 2:**
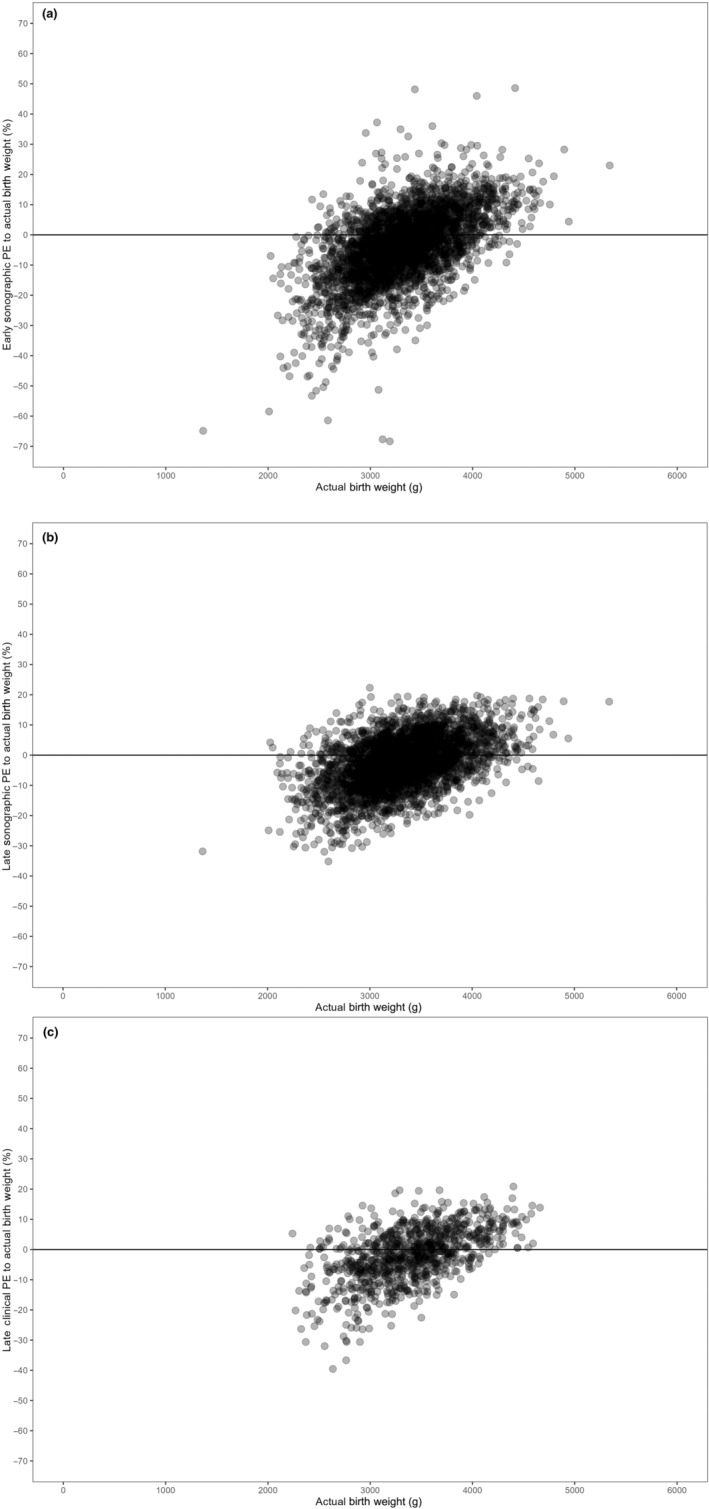
Comparison of weight prediction percentage errors (PE) against actual birth weights. Bland–Altman plots comparing PE of weight predictions from early and late weight estimations and actual birth weights. (a) Early sonographic weight estimations, (b) late sonographic weight estimations, and (c) late clinical weight estimations, all in comparison to actual birth weights (grams).

Figure 2 illustrates the percentage errors in weight estimations as compared with the actual birth weights across the range of fetal actual birth weights. The graphs plot these errors, showing the spread and distribution of discrepancies between estimated and actual weights. Each data point represents a direct observation from the cohort, arranged to visually depict any variations across the observed birth weight spectrum.

Weight estimation accuracy in various maternal and fetal subgroups is detailed in Table 3. In the subgroup of maternal obesity, clinical weight estimates were significantly less accurate than in non‐obese women (*P* < 0.001), although both early and late sonographic estimates showed no notable difference (*P* = 0.081 and *P* = 0.070, respectively). For women with pregestational or gestational diabetes, early sonographic weight estimates were significantly more accurate compared with those without gestational diseases (*P* < 0.001), but this pattern did not extend to late sonographic or clinical estimates (*P* = 0.309 and *P* = 0.904, respectively). In cases of small‐for‐gestational‐age (SGA) or large‐for‐gestational‐age (LGA) fetuses, all weight estimation methods were markedly less precise than in fetuses with birth weights between the 10th and 90th centiles (*P* < 0.001).

**TABLE 3:** Measures of accuracy in birth weight prediction in special subgroups of interest.^a^

	MPE		Random error		MPE ± 95% CI^b^, ^c^		Mean absolute percentage error	
	Subgroup of interest	Reference population^d^	Subgroup of interest	Reference population^d^	Subgroup of interest	Reference population^d^	Subgroup of interest	Reference population^d^
Maternal obesity^e^ (*n* = 313)								
Early sonographic projected weight	−2.54	−3.84	11.56	11.67	−2.54 ± 22.66	−3.84 ± 22.87	9.03	9.34
Late sonographic projected weight	−3.38	−2.53	7.64	8.54	−3.38 ± 14.97	−2.53 ± 16.74	6.52	7.04
Late clinical projected weight	−5.85	−1.66	8.86	8.7	**−5.85 ± 17.37**	**−1.66 ± 17.05**	7.85	6.91
Pregestational/gestational diabetes (*n* = 340)								
Early sonographic projected weight	−1.31	−3.94	12.23	11.64	**−1.31 ± 23.97**	**−3.94 ± 22.81**	9.26	9.33
Late sonographic projected weight	−2.08	−2.55	8.52	8.54	−2.08 ± 16.7	−2.55 ± 16.74	6.96	7.01
Late clinical projected weight	−1.6	−1.94	8.05	8.91	−1.6 ± 15.78	−1.94 ± 17.46	6.17	7.03
SGA^f^ (*n* = 318)								
Early sonographic projected weight	−18.35	−3.85	13.15	9.95	**−18.35 ± 25.77**	**−3.85 ± 19.5**	19.15	8.22
Late sonographic projected weight	−10.36	−2.71	8.92	7.88	**−10.36 ± 17.48**	**−2.71 ± 15.44**	11.32	6.6
Late clinical projected weight	−11.58	−2.51	9.97	7.97	**−11.58 ± 19.54**	**−2.51 ± 15.62**	12.5	6.44
LGA^g^ (*n* = 492)								
Early sonographic projected weight	6.71	−3.85	9.07	9.95	**6.71 ± 17.78**	**−3.85 ± 19.5**	9.09	8.22
Late sonographic projected weight	3.73	−2.71	7.01	7.88	**3.73 ± 13.73**	**−2.71 ± 15.44**	6.49	6.6
Late clinical projected weight	5.08	−2.51	6.05	7.97	**5.08 ± 11.86**	**−2.51 ± 15.62**	6.56	6.44

Abbreviations: CI, confidence interval; LGA, large for gestational age; MPE, mean percentage error; SGA, small for gestational age;

^a^ The systematic error is the MPE of the projected weights, calculated as the mean of (birth weight – sonographic or clinical projected weight)/birth weight × 100. Negative values represent overestimation of actual birth weight and positive values represent underestimations. Random error is the standard deviation of the mean percentage error.

^b^ According to the limits of agreement method.

^c^ MPE were compared between projected weights from special subgroups of interest and compared with the reference populations using Wilcoxon signed ranks test, all mean values which were significantly different (*P* < 0.001) are marked in bold. In the subgroup of maternal obesity, a significant difference (*P* < 0.001) was found only in late clinical projected weights, *P* values of early and late sonographic estimations were *P* = 0.081 and *P* = 0.070, respectively. In the subgroup of maternal diabetes, a significant difference (*P* < 0.001) was only found in early sonographic weight estimations, *P* values of late sonographic and clinical estimations were *P* = 0.309 and *P* = 0.904, respectively. In the subgroup of SGA and LGA fetuses, all weight estimations were significantly different when compared with the reference population (*P* < 0.001).

^d^ Reference populations: subgroup of maternal obesity (*n* = 313) was compared with a subgroup of body mass index less than 30 (calculated as weight in kilograms divided by the square of height in meters) (*n* = 2576); subgroup of pregestational/gestational diabetes (*n* = 340) was compared with a subgroup with no diabetes (*n* = 3209), subgroups of SGA (*n* = 318) and LGA (*n* = 492) fetuses were compared with a subgroup of fetuses whose actual birth weights were between the 10th and 90th centiles (*n* = 2739).

^e^ Maternal obesity defined as body mass index >30 (calculated as weight in kilograms divided by the square of height in meters).

^f^ Small for gestational age was actual birth weight <10th centile.

^g^ Large for gestational age was actual birth weight >90th centile.

## 4 DISCUSSION

In this large database of prenatal sonographic and clinical examinations, weight estimations conducted at term were more accurate in predicting actual birth weight in comparison to sonographic weight estimations from earlier third trimester. Furthermore, a positive trend in accurate weight estimations was demonstrated as the pregnancy progressed beyond term. Most weight predictions, in all timings and methods tested, tended to overestimate the actual birth weight, with the smallest overestimation proportion in late clinical estimations. In patients with obesity, clinical weight estimations were found to be less accurate when compared with non‐obese mothers, and in SGA and LGA fetuses, all weight estimations were less accurate when compared with fetuses born at weights between the 10th and 90th centiles.

Most previous studies on healthy singleton pregnancies demonstrated higher accuracy in weight estimations when conducted during the late third trimester, when compared with earlier estimations. This finding has been consistent across different populations, including Greek and Australian cohorts.^11^, ^13^, ^17^ However, the study by Pressman et al.,^12^ which focused on an American population, presents an exception by showing an advantage to sonograms between 34^+0^ and 36^+9^ weeks of pregnancy, when compared with 37 weeks and beyond. However, the latter study was relatively small and included only 138 patients. In our data, the lowest systematic and random errors were produced using late clinical and sonographic estimations, with MPE of −1.9 and −2.5, and random errors of 8.9 and 8.5, respectively. These results are consistent with MPE within the 5% level of accuracy sought in practice.^2^, ^3^ No significant difference was demonstrated between late sonographic and clinical estimations, in accordance with the literature.^18^ The tendency to overestimate fetal birth weights is consistent with the conclusions of the systematic review by Milner and Arezina,^3^ which described most sonographic ultrasound calculations of fetal weights to be larger than actual birth weights.

The finding of increased accuracy of sonographic weight estimations from each gestational week as the pregnancy advanced past term is supported by Oliver et al.,^17^ who showed the lowest systematic error with scans conducted within 2 weeks from delivery, when compared with larger time intervals. This observation can be linked to a re‐evaluation of commonly held beliefs regarding fetal growth. Contrary to the widespread notion that normal fetuses adhere to centile growth curves with near‐perfect accuracy, this finding suggests that this may not universally hold true. This casts doubt on the reliability of the projection formula, commonly used on the base of earlier third‐trimester weight estimations. Additionally, it warrants consideration that fetuses classified as normal during pregnancy may not be entirely exempt from episodes of growth restriction or acceleration, thereby potentially explaining the improved accuracy of weight estimations as the pregnancy progresses.

In subgroup analysis, no notable disparity was demonstrated in sonographic measurements taken early and late in pregnancy when compared between women with and without obesity. This observation persists despite the acknowledged challenges in obtaining ultrasound measurements in overweight women, as discussed in Paladini's review.^19^ Previous studies have similarly reported no significant differences.^20^, ^21^ However, the accuracy of clinical weight estimations for pregnant women with obesity was markedly lower compared with their non‐obese counterparts, aligning with earlier research.^22^ A possible explanation for the diminished accuracy of clinical weight predictions in patients with obesity is their larger physical size, which obstructs the precision of clinical assessments. Consequently, despite the difficulties associated with sonographic predictions in this population, they still appear to represent a more effective method for reducing discrepancies in weight estimations, when compared with clinical estimation methods.

Despite the association between gestational and pregestational diabetes with higher rates of LGA newborns,^23^ previous research has not demonstrated a significant difference in the accuracy of sonographic measurements between this group and their non‐diabetic counterparts,^7^, ^24^, ^25^ which is consistent with our findings. Furthermore, our results are supported by a study by Moore et al.,^14^ which found increased precision in later sonographic assessments, even in women with diabetes. Notably, our study uncovers improved precision in early sonographic evaluations in patients with maternal diabetes, when compared with their counterparts. This enhancement, predominantly seen in systematic errors rather than random errors, could be linked to clinicians’ awareness of the patients’ diabetic status, leading them to anticipate larger fetal sizes and adjust their weight estimations accordingly. This tendency towards larger assessments and enhanced accuracy in weight estimations continued into later sonographic and clinical assessments, but the difference was not statistically significant. This suggests that although initial adjustments based on diabetic status may influence earlier measurements, the cumulative impact on the accuracy of subsequent assessments throughout pregnancy remains minimal. Therefore, our data suggest that no special adjustment in weight estimation techniques should be implied in this population.

In fetuses born with a birth weight below the 10th centile, all weight estimations were significantly less accurate when compared with those within the 10th–90th centile range. Consistent with observations in the broader study population, weight estimations from the later third trimester demonstrated improved accuracy, addressing both systematic and random errors effectively. This observation aligns with findings from a recent review by Mustafa et al.,^26^ which highlighted better predictive accuracy for SGA neonates from sonograms conducted at 36 weeks of gestation as opposed to 32 weeks. The late sonographic weight assessments in our study were conducted at more advanced stages of pregnancy, yet the trend of increasing accuracy in weight estimations as the pregnancy progresses was evident in the outcomes of both studies.

Unlike the wider study population and the specific subgroups examined, weight estimations for LGA fetuses were more likely to underestimate the actual birth weight during both early and late third trimester. This observation is consistent with several previous studies^2^, ^6^, ^27^ although research by Zafman et al.^28^ presents an exception, indicating overestimation in the majority of fetuses with macrosomia. Later weight assessments in LGA fetuses tended for improved accuracy in forecasting actual birth weights, mirroring the pattern observed in the broader study cohort. However, the consistency of this result remains uncertain in existing literature.^13^, ^29^

The observation of larger disparities in weight estimations of SGA and LGA fetuses highlights the already recognized challenges in predicting birth weight accurately in these groups and emphasizes the necessity for improved screening and diagnostic approaches. Finally, given our findings of birth weight underestimation in LGA fetuses, we advocate for a heightened awareness of macrosomia in this group, especially in assessments carried out in the late third trimester. Given the variability of these findings in existing literature, additional research is warranted to further clarify this issue.

Our study contains several strengths, including a large database, comprising a wide cohort of singleton gestations, with repeated weight estimations performed on the same fetuses throughout gestation. This includes a broad range of cases with specific maternal and fetal subpopulations. Given the importance and the implications of weight prediction in antenatal care, we expect our results to enrich our understanding of the advantages and limitations associated with weight prediction techniques. Consequently, leading to enhancements in obstetric care and clinical decision making processes.

However, our study carries several limitations. The retrospective design introduces the potential for reporting bias, as our data were derived from electronic medical records, which are susceptible to human error. Additionally, our research population consisted exclusively of Israeli women. While this group includes a wide variety of ethnicities, it may limit the generalizability of our findings to other populations worldwide. Despite the consistency of our results with existing literature, these factors highlight the need for future research to confirm and extend our findings.

In conclusion, in this retrospective cohort study, weight predictions later in pregnancy were significantly more precise in forecasting actual birth weights, when compared with earlier weight estimations. Therefore, we recommend that projected birth weight should not rely solely on early third‐trimester sonograms. Instead, assessment of fetal weight should be conducted closer to the time of delivery, to improve accuracy. Our findings emphasize the difficulties in accurately predicting weights for SGA and LGA fetuses, with a noted tendency to underestimate birth weights in LGA cases, unlike in the general population. In obese women, later sonographic evaluations proved more accurate than clinical methods. However, for women with diabetes, no significant differences were found in later weight estimations, indicating no need for adjustments in this group.

## AUTHOR CONTRIBUTIONS

SR‐S wrote the manuscript writing and contributed to data analysis and collection; WA contributed to study design and planning, and to revision of the manuscript; AZe contributed to data collection and analysis; NC and OL contributed to revision of the manuscript; AZi contributed to study design and planning and to revision of manuscript, and was research supervisor.

## CONFLICTS OF INTEREST STATEMENT

The authors have no conflicts of interest.

## Data Availability

The data that support the findings of this study are available from the corresponding author upon reasonable request.
